# Crystal structure of a new tripotassium hexa­nickel iron hexa­phosphate

**DOI:** 10.1107/S2056989019002706

**Published:** 2019-02-26

**Authors:** Said Ouaatta, Abderrazzak Assani, Mohamed Saadi, Lahcen El Ammari

**Affiliations:** a Laboratoire de Chimie Appliquée des Matŕiaux, Centre des Sciences des Matériaux, Faculty of Sciences, Mohammed V University in Rabat, Avenue Ibn Batouta, BP 1014, Rabat, Morocco

**Keywords:** crystal structure, crystal growth, β-xenophyllite structure, K_3_Ni_6_Fe(PO_4_)_6_, X-ray diffraction, othophosphate, solid-state reaction synthesis

## Abstract

A new potassium-nickel iron phosphate, K_3_Ni_6_Fe(PO_4_)_6_, has been synthesized by solid-state reaction and structurally characterized by single-crystal X-ray diffraction and qualitative energy dispersive X-ray spectroscopy (EDS) analysis. The structure is built up by [FeO_6_], [PO_4_], and [NiO_6_] coordination polyhedra, which are linked to each other by edge and corner sharing to form zigzag layers parallel to the *ab* plane. These layers are inter­connected by [PO_4_] tetra­hedra and [NiO_6_] octa­hedra *via* common corners, leading to a three-dimensional framework delimiting large channels running along the [100] direction in which the K^+^ cations are localized.

## Abstract 2

K_3_Ni_6_Fe(PO_4_)_6_ has been synthesized by solid-state reaction and structurally characterized by single-crystal X-ray diffraction. Its structure is built up by [PO_4_] tetra­hedra and [FeO_6_] and [NiO_6_] octa­hedra linked to each other by edge or corner sharing, leading to a three-dimensional framework delimiting tunnels along the [100] direction in which the K^+^ cations are localized.

## Chemical context   

Iron-based phosphates are widely studied materials today. They present a promising field for various applications such as electronics (Saw *et al.*, 2014 ▸), ferroelectrics (Lazoryak *et al.*, 2004 ▸), magnetic materials (Hatert *et al.*, 2004 ▸; Essehli *et al.*, 2015 ▸) and catalytic processes (Moffat, 1978 ▸). The introduction of alkali metals into these phosphates materials can be of great inter­est to improve the ion-conduction properties for applications in rechargeable alkaline batteries (La Parola *et al.*, 2018 ▸; Orikasa *et al.*, 2016 ▸). The present work is part of our activity devoted particularly to the investigation of new materials based on phosphates belonging to the *A*_2_O–*M*O–Fe_2_O_3_–P_2_O_5_ (*A* = an alkali metal; *M* = divalent cation) quaternary system, which could have inter­esting ionic conductivity or magnetic proprieties. We report herein on the synthesis and structural characterization by single crystal X-ray diffraction of a new potassium nickel iron phosphate with formula K_3_Ni_6_Fe(PO_4_)_6_.

## Structural commentary   

**Figure 1:**
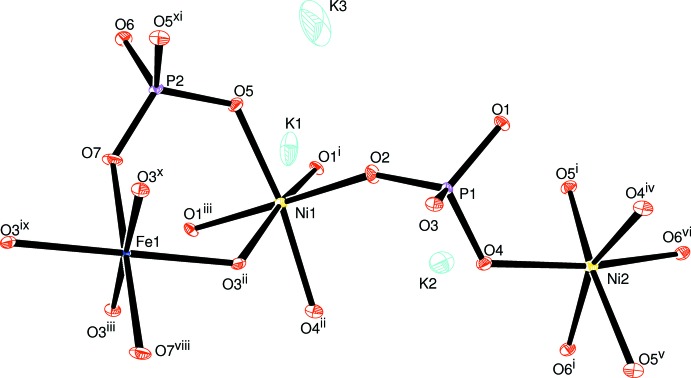
Mol­ecular structure of the title compound with the atom-labelling scheme. Displacement ellipsoids are drawn at the 50% probability level. Symmetry codes: (i) −*x* + 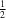, −*y* + 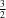, −*z* + 1; (ii) −*x* + 1, *y*, −*z* + 1; (iii) *x*, *y*, *z* − 1; (iv) −*x* + 1, *y*, −*z* + 2; (v) *x* + 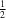, −*y* + 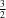, *z* + 1; (vi) *x* + 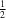, *y* + 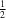, *z* + 1; (vii) −*x* + 1, −*y* + 1, −*z*; (viii) *x*, −*y* + 1, *z* − 1; (ix) −*x* + 1, −*y* + 1, −*z* + 1; (*x*) *x*, −*y* + 1, *z*; (xi) *x*, *y*, *z* + 1; (xii) −*x* + 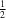, −*y* + 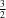, −*z*; (xiii) −*x*, −*y* + 1, −*z*; (xiv) *x*, −*y* + 2, *z*.

**Figure 2:**
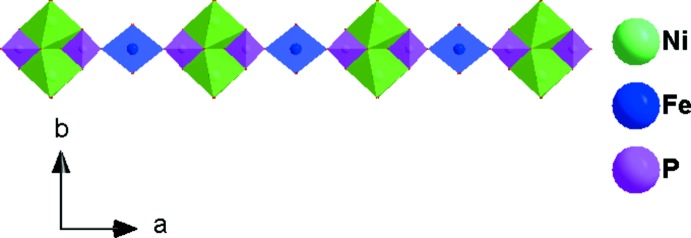
A chain formed by sharing edges and corners of [Ni2_2_O_10_] dimers, [P2O_4_] tetra­hedra and [FeO_6_] octa­hedra along the [100] direction

**Figure 3:**
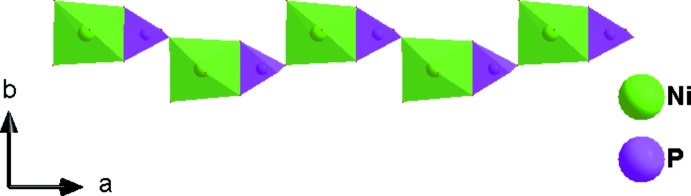
Corner- and edge-sharing [P1O_4_] tetra­hedra and [Ni1O_6_] octa­hedra forming a zigzag shape chain running parallel to [100]

**Figure 4:**
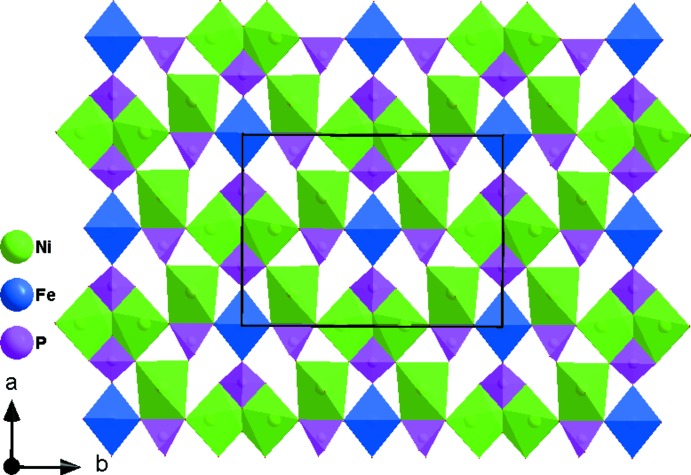
View along the *c* axis of corner- and edge-sharing [PO_4_] tetra­hedra and [NiO_6_] octa­hedra forming a layer parallel to the *ab* plane.

**Figure 5:**
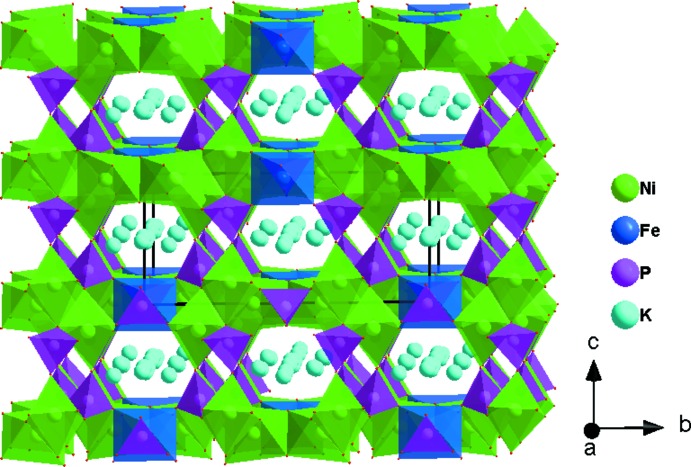
Polyhedral representation of the crystal structure of K_3_Ni_6_Fe(PO_4_)_6_ showing large tunnels running along the [100] direction that contain the K^+^ cations.

The asymmetric unit of the title compound, K_3_Ni_6_Fe(PO_4_)_6_, consists of two [NiO_6_] octa­hedra, one [FeO_6_] octa­hedron, two [PO_4_] tetra­hedra, and three K atoms, as shown in Fig. 1 ▸. One Ni^2+^, Fe^3+^, P^5+^, two K^+^ cations and two of the seven oxygen atoms lie on special positions. The Ni2 atom occupies Wyckoff position 4*g* (2), the Fe atom is localized on the 2*a* (2/*m*) Wyckoff position, P2, K1, K3, O6 and O7 are positioned on 4*i* (*m*) sites. The octa­hedral coordination sphere of the nickel(II) cation is more distorted than that of the iron(III) atom, with average <Ni—O> distances of 2.066 and 2.119 Å for Ni1 and Ni2, respectively. The mean <P—O> distance in the two PO_4_ tetra­hedra is equal to 1.547 Å for P1 and 1.543 Å for P2. The Fe atoms are coordinated octa­hedrally with an average <Fe—O> distance of 2.038 Å. The structure of the title compound is built up from two types of nickel sites and one iron site, each with an octa­hedral coordination environment, [Ni1O_6_], [Ni2O_6_] and [FeO_6_], besides two independent phosphor tetra­hedra [P1O_4_] and [P2O_4_]. Edge-sharing [Ni2O_6_] octa­hedra build up a dimeric [Ni2_2_O_10_] unit. Two [P2O_6_] octa­hedra are connected to the [Ni2_2_O_10_] dimer by sharing edges to form an [Ni(2)_2_P(2)_2_O_12_] unit, which alternates with an [FeO_6_] octa­hedron to establish an infinite chain along the [100] direction (Fig. 2 ▸). In addition, the association between the [P1O_4_] tetra­hedra and the [Ni1O_6_] octa­hedra by means of edge-sharing allows the formation of a zigzag chain running parallel to the [100] direction. Each of the P1O_4_ tetra­hedra and Ni1O_6_ octa­hedra, both belonging to the same layer, share vertices with Ni1O_6_ and P1O_4_, respectively, of the adjacent one (Fig. 3 ▸). The two types of chain linkages lead to the formation of layers parallel to the *ab* plane (Fig. 4 ▸). One vertex of an Ni1O_6_ octa­hedron belonging to one layer is shared with a P1O_4_ vertex of the neighbouring layer. This configuration leads to a three-dimensional centrosymmetric framework, delimiting hexa­gonal tunnels along the [100] direction, in which the K^+^ cations are located (Fig. 5 ▸). The potassium cations are distributed over three independent crystallographic positions with partial occupancies

## Database survey   

The investigated compound is a new member of the β-xenophyllite family that includes Na_4_Ni_7_(PO_4_)_6_ (Moring & Kostiner, 1986 ▸), Na_4_Co_7_(PO_4_)_6_ (Kobashi *et al.*, 1998 ▸), K_4_Ni_7_(AsO_4_)_6_ (Ben Smail *et al.*, 1999 ▸), Na_4_Co_5.63_Al_0.91_(AsO_4_)_6_ (Marzouki *et al.*, 2010 ▸), Na_4_Li_0.62_Co_5.67_Al_0.71_(AsO_4_)_6_ (Marzouki *et al.*, 2013 ▸), Ag_4_Co_7_(AsO_4_)_6_ (Marzouki *et al.*, 2014 ▸) and Na_4_Co_7_(AsO_4_)_6_ (Ben Smida *et al.*, 2016 ▸). The phosphates of these compounds crystallize in the non-centrosymmetric *Cm* space group while the arsenates adopt the *C*2/*m* space group.

## Synthesis and crystallization   

Single crystals of K_3_Ni_6_Fe(PO_4_)_6_ were prepared by solid-state reaction in air. A mixture of K_2_CO_3_, Ni(NO_3_)_2_·6H_2_O, Fe(NO_3_)_3_·9H_2_O and H_3_PO_4_ (85 wt.%) reagents with a K:Ni:Fe:P molar ratio of 2:2:1:3 was dissolved in 50 mL of distilled water. The resulting solution was stirred without heating for 24 h and was subsequently evaporated to dryness at 343 K. The obtained dry residue was progressively heated in a platinum crucible up to 673 K in order to eliminate volatile products. In a second step, the powder was homogenized in an agate mortar and then progressively heated to 1303 K. Kept at this temperature for 2 h, the reaction mixture then underwent slow cooling at a rate of 5 Kh^−1^ to 1103 K and then to room temperature with the furnace inertia. After washing with distilled water, the obtained crystals were brown with block-type shape. A qualitative EDX analysis (energy dispersive X-ray spectroscopy) detected the presence of the expected chemical elements corresponding to K, Ni, Fe, P and O atoms (see Fig. 6).

## Refinement   

Crystal data, data collection and structure refinement details of K_3_Ni_6_Fe(PO_4_)_6_ are summarized in Table 1 ▸. The highest peak and the deepest hole in the final Fourier map are at 0.71 and 0.59 Å, respectively, from atom K2.

## Supplementary Material

Crystal structure: contains datablock(s) I. DOI: 10.1107/S2056989019002706/vn2144sup1.cif

Structure factors: contains datablock(s) I. DOI: 10.1107/S2056989019002706/vn2144Isup2.hkl

CCDC reference: 1898755

Additional supporting information:  crystallographic information; 3D view; checkCIF report

## Figures and Tables

**Table 1:** Experimental details

Crystal data	
Chemical formula	K_3_Ni_6_Fe(PO_4_)_6_
*M*_r_	1095.23
Crystal system, space group	Monoclinic, *C*2/*m*
Temperature (K)	296
*a*, *b*, *c* (Å)	10.6853 (4), 14.1009 (5), 6.5481 (2)
β (°)	103.842 (1)
*V* (Å^3^)	957.97 (6)
*Z*	2
Radiation type	Mo *K*α
μ (mm^−1^)	7.79
Crystal size (mm)	0.36 × 0.27 × 0.20

Data collection	
Diffractometer	Bruker D8 VENTURE Super DUO
Absorption correction	Multi-scan (*SADABS*; Krause *et al.*, 2015 ▸)
*T*_min_, *T*_max_	0.638, 0.746
No. of measured, independent and observed [*I* > 2σ(*I*)] reflections	13870, 1981, 1891
*R*_int_	0.021
(sin θ/λ)_max_ (Å^−1^)	0.781

Refinement	
*R*[*F* ^2^ > 2σ(*F* ^2^)], *wR*(*F* ^2^), *S*	0.024, 0.062, 1.07
No. of reflections	1981
No. of parameters	112
Δρ_max_, Δρ_min_ (e Å^−3^)	2.34, −1.16

Computer programs: *APEX3* and *SAINT-Plus* (Bruker, 2016 ▸), *SHELXT2014* (Sheldrick, 2015*a* ▸), *SHELXL2016* (Sheldrick, 2015*b* ▸), *ORTEP-3 for Windows* (Farrugia, 2012 ▸), *DIAMOND* (Brandenburg, 2006 ▸) and *publCIF* (Westrip, 2010 ▸).

## supplementary crystallographic information

### Crystal data

**Table:**

K_3_Ni_6_Fe(PO_4_)_6_	*F*(000) = 1066
*M**_r_* = 1095.23	*D*_x_ = 3.797 Mg m^−^^3^
Monoclinic, *C*2/*m*	Mo *K*α radiation, λ = 0.71073 Å
*a* = 10.6853 (4) Å	Cell parameters from 1981 reflections
*b* = 14.1009 (5) Å	θ = 2.4–33.7°
*c* = 6.5481 (2) Å	µ = 7.79 mm^−^^1^
β = 103.842 (1)°	*T* = 296 K
*V* = 957.97 (6) Å^3^	Block, brown
*Z* = 2	0.36 × 0.27 × 0.20 mm

### Data collection

**Table:**

Bruker D8 VENTURE Super DUO diffractometer	1981 independent reflections
Radiation source: INCOATEC IµS micro-focus source	1891 reflections with *I* > 2σ(*I*)
HELIOS mirror optics monochromator	*R*_int_ = 0.021
Detector resolution: 10.4167 pixels mm^-1^	θ_max_ = 33.7°, θ_min_ = 2.4°
φ and ω scans	*h* = −14→16
Absorption correction: multi-scan (SADABS; Krause *et al.*, 2015)	*k* = −22→22
*T*_min_ = 0.638, *T*_max_ = 0.746	*l* = −10→10
13870 measured reflections	

### Refinement

**Table:**

Refinement on *F*^2^	112 parameters
Least-squares matrix: full	0 restraints
*R*[*F*^2^ > 2σ(*F*^2^)] = 0.024	*w* = 1/[σ^2^(*F*_o_^2^) + (0.0262*P*)^2^ + 6.1957*P*] where *P* = (*F*_o_^2^ + 2*F*_c_^2^)/3
*wR*(*F*^2^) = 0.062	(Δ/σ)_max_ = 0.001
*S* = 1.07	Δρ_max_ = 2.34 e Å^−^^3^
1981 reflections	Δρ_min_ = −1.16 e Å^−^^3^

### Special details

**Table:**

Geometry. All esds (except the esd in the dihedral angle between two l.s. planes) are estimated using the full covariance matrix. The cell esds are taken into account individually in the estimation of esds in distances, angles and torsion angles; correlations between esds in cell parameters are only used when they are defined by crystal symmetry. An approximate (isotropic) treatment of cell esds is used for estimating esds involving l.s. planes.

### Fractional atomic coordinates and isotropic or equivalent isotropic displacement parameters (Å^2^)

**Table:**

	*x*	*y*	*z*	*U*_iso_*/*U*_eq_	Occ. (<1)
Ni1	0.33034 (2)	0.69342 (2)	0.18429 (4)	0.00599 (6)	
Ni2	0.500000	0.87799 (2)	1.000000	0.00683 (7)	
Fe1	0.500000	0.500000	0.000000	0.00327 (9)	
K1	0.40983 (18)	0.500000	0.4730 (2)	0.0200 (3)	0.472
K2	0.38224 (13)	0.90311 (11)	0.48618 (19)	0.0242 (3)	0.417
K3	0.1849 (7)	0.500000	0.4893 (8)	0.0560 (19)	0.192
P1	0.41063 (4)	0.69522 (3)	0.72442 (7)	0.00485 (8)	
P2	0.19569 (6)	0.500000	−0.01061 (10)	0.00557 (11)	
O1	0.31025 (13)	0.70594 (10)	0.8587 (2)	0.0072 (2)	
O2	0.34525 (14)	0.68172 (11)	0.4959 (2)	0.0102 (2)	
O3	0.50488 (13)	0.60977 (10)	0.7970 (2)	0.0069 (2)	
O4	0.50525 (13)	0.78185 (10)	0.7692 (2)	0.0082 (2)	
O5	0.19430 (13)	0.58983 (10)	0.1265 (2)	0.0085 (2)	
O6	0.06136 (18)	0.500000	−0.1681 (3)	0.0087 (3)	
O7	0.30728 (19)	0.500000	−0.1148 (3)	0.0103 (3)	

### Atomic displacement parameters (Å^2^)

**Table:**

	*U*^11^	*U*^22^	*U*^33^	*U*^12^	*U*^13^	*U*^23^
Ni1	0.00441 (10)	0.00697 (10)	0.00638 (10)	0.00038 (7)	0.00088 (7)	−0.00015 (7)
Ni2	0.00519 (13)	0.00602 (14)	0.00944 (14)	0.000	0.00207 (10)	0.000
Fe1	0.00218 (18)	0.00293 (18)	0.00478 (19)	0.000	0.00099 (15)	0.000
K1	0.0413 (9)	0.0080 (5)	0.0103 (5)	0.000	0.0055 (5)	0.000
K2	0.0259 (6)	0.0332 (7)	0.0122 (4)	−0.0107 (5)	0.0018 (4)	0.0082 (4)
K3	0.056 (4)	0.100 (6)	0.0100 (17)	0.000	0.005 (2)	0.000
P1	0.00395 (17)	0.00570 (18)	0.00483 (18)	0.00067 (13)	0.00093 (14)	−0.00035 (13)
P2	0.0033 (2)	0.0049 (2)	0.0082 (3)	0.000	0.00071 (19)	0.000
O1	0.0055 (5)	0.0095 (5)	0.0072 (5)	0.0022 (4)	0.0028 (4)	0.0009 (4)
O2	0.0091 (6)	0.0162 (6)	0.0048 (5)	0.0011 (5)	0.0003 (4)	0.0002 (4)
O3	0.0049 (5)	0.0070 (5)	0.0089 (5)	0.0014 (4)	0.0018 (4)	0.0012 (4)
O4	0.0069 (5)	0.0072 (5)	0.0110 (6)	−0.0012 (4)	0.0031 (4)	−0.0022 (4)
O5	0.0071 (5)	0.0067 (5)	0.0120 (6)	−0.0012 (4)	0.0027 (4)	−0.0031 (4)
O6	0.0045 (7)	0.0124 (8)	0.0081 (8)	0.000	−0.0005 (6)	0.000
O7	0.0056 (7)	0.0088 (8)	0.0181 (9)	0.000	0.0060 (7)	0.000

### Geometric parameters (Å, º)

**Table:**

Ni1—O2	2.0153 (14)	K1—O7^xi^	3.146 (3)
Ni1—O5	2.0314 (14)	K2—O4	2.631 (2)
Ni1—O1^i^	2.0366 (13)	K2—O6^xii^	2.677 (2)
Ni1—O3^ii^	2.0984 (14)	K2—O2^i^	2.737 (2)
Ni1—O1^iii^	2.0988 (14)	K2—O5^i^	2.8470 (19)
Ni1—O4^ii^	2.1161 (14)	K2—O4^ii^	2.851 (2)
Ni2—O4	2.0411 (14)	K2—O6^vi^	2.929 (2)
Ni2—O4^iv^	2.0411 (14)	K2—O1^i^	3.0776 (19)
Ni2—O5^v^	2.0928 (14)	K2—O7^xii^	3.086 (2)
Ni2—O5^i^	2.0928 (14)	K2—O2	3.149 (2)
Ni2—O6^i^	2.2241 (13)	K3—O7^xi^	2.609 (6)
Ni2—O6^vi^	2.2241 (13)	K3—O5	2.715 (5)
Fe1—O7	2.016 (2)	K3—O5^x^	2.715 (5)
Fe1—O7^vii^	2.016 (2)	K3—O6^xi^	2.862 (6)
Fe1—O3^viii^	2.0490 (13)	K3—O6^xiii^	2.949 (7)
Fe1—O3^ii^	2.0490 (13)	K3—O2	3.077 (4)
Fe1—O3^ix^	2.0490 (13)	K3—O2^x^	3.077 (4)
Fe1—O3^iii^	2.0490 (13)	P1—O2	1.5042 (15)
K1—O3	2.6263 (18)	P1—O1	1.5482 (14)
K1—O3^x^	2.6264 (18)	P1—O4	1.5679 (14)
K1—O2	2.6673 (16)	P1—O3	1.5694 (14)
K1—O2^x^	2.6673 (16)	P2—O7	1.509 (2)
K1—O3^ix^	2.6691 (19)	P2—O6	1.554 (2)
K1—O3^ii^	2.6691 (19)	P2—O5^x^	1.5546 (14)
K1—O5	3.091 (2)	P2—O5	1.5546 (14)
K1—O5^x^	3.091 (2)		
			
O2—Ni1—O5	90.55 (6)	O3^ix^—K1—O5	93.66 (6)
O2—Ni1—O1^i^	94.23 (6)	O3^ii^—K1—O5	65.70 (5)
O5—Ni1—O1^i^	90.23 (6)	O3—K1—O5^x^	155.60 (8)
O2—Ni1—O3^ii^	91.89 (6)	O3^x^—K1—O5^x^	115.32 (5)
O5—Ni1—O3^ii^	99.19 (5)	O2—K1—O5^x^	106.12 (6)
O1^i^—Ni1—O3^ii^	168.72 (5)	O2^x^—K1—O5^x^	59.36 (5)
O2—Ni1—O1^iii^	178.70 (6)	O3^ix^—K1—O5^x^	65.70 (5)
O5—Ni1—O1^iii^	88.65 (6)	O3^ii^—K1—O5^x^	93.66 (6)
O1^i^—Ni1—O1^iii^	84.74 (6)	O5—K1—O5^x^	48.38 (6)
O3^ii^—Ni1—O1^iii^	89.25 (5)	O3—K1—O7^xi^	56.89 (5)
O2—Ni1—O4^ii^	92.33 (6)	O3^x^—K1—O7^xi^	56.89 (5)
O5—Ni1—O4^ii^	169.40 (5)	O2—K1—O7^xi^	78.69 (5)
O1^i^—Ni1—O4^ii^	99.72 (5)	O2^x^—K1—O7^xi^	78.69 (5)
O3^ii^—Ni1—O4^ii^	70.53 (5)	O3^ix^—K1—O7^xi^	144.33 (3)
O1^iii^—Ni1—O4^ii^	88.64 (5)	O3^ii^—K1—O7^xi^	144.33 (3)
O4—Ni2—O4^iv^	96.75 (8)	O5—K1—O7^xi^	106.17 (7)
O4—Ni2—O5^v^	103.69 (6)	O5^x^—K1—O7^xi^	106.17 (7)
O4^iv^—Ni2—O5^v^	92.95 (5)	O4—K2—O6^xii^	135.41 (8)
O4—Ni2—O5^i^	92.95 (5)	O4—K2—O2^i^	89.14 (6)
O4^iv^—Ni2—O5^i^	103.69 (6)	O6^xii^—K2—O2^i^	128.60 (7)
O5^v^—Ni2—O5^i^	154.96 (8)	O4—K2—O5^i^	66.22 (5)
O4—Ni2—O6^i^	160.71 (6)	O6^xii^—K2—O5^i^	147.28 (7)
O4^iv^—Ni2—O6^i^	94.81 (6)	O2^i^—K2—O5^i^	61.95 (5)
O5^v^—Ni2—O6^i^	91.06 (6)	O4—K2—O4^ii^	79.26 (7)
O5^i^—Ni2—O6^i^	69.28 (6)	O6^xii^—K2—O4^ii^	69.19 (5)
O4—Ni2—O6^vi^	94.80 (6)	K2^xiv^—K2—O4^ii^	126.86 (4)
O4^iv^—Ni2—O6^vi^	160.71 (6)	O2^i^—K2—O4^ii^	105.45 (6)
O5^v^—Ni2—O6^vi^	69.28 (6)	O5^i^—K2—O4^ii^	142.65 (7)
O5^i^—Ni2—O6^vi^	91.06 (6)	O4—K2—O6^vi^	68.58 (5)
O6^i^—Ni2—O6^vi^	78.66 (8)	O6^xii^—K2—O6^vi^	97.82 (7)
O7—Fe1—O7^vii^	180.0	O2^i^—K2—O6^vi^	126.41 (7)
O7—Fe1—O3^viii^	86.58 (5)	O5^i^—K2—O6^vi^	64.47 (5)
O7^vii^—Fe1—O3^viii^	93.42 (5)	O4^ii^—K2—O6^vi^	116.35 (6)
O7—Fe1—O3^ii^	93.42 (5)	O4—K2—O1^i^	109.01 (6)
O7^vii^—Fe1—O3^ii^	86.58 (5)	O6^xii^—K2—O1^i^	85.38 (5)
O3^viii^—Fe1—O3^ii^	180.00 (8)	O2^i^—K2—O1^i^	50.84 (4)
O7—Fe1—O3^ix^	93.42 (5)	O5^i^—K2—O1^i^	112.77 (6)
O7^vii^—Fe1—O3^ix^	86.58 (5)	O4^ii^—K2—O1^i^	64.63 (5)
O3^viii^—Fe1—O3^ix^	81.87 (8)	O6^vi^—K2—O1^i^	176.79 (6)
O3^ii^—Fe1—O3^ix^	98.13 (8)	O4—K2—O7^xii^	165.00 (7)
O7—Fe1—O3^iii^	86.58 (5)	O6^xii^—K2—O7^xii^	52.30 (6)
O7^vii^—Fe1—O3^iii^	93.42 (5)	O2^i^—K2—O7^xii^	78.76 (5)
O3^viii^—Fe1—O3^iii^	98.13 (8)	O5^i^—K2—O7^xii^	114.35 (7)
O3^ii^—Fe1—O3^iii^	81.87 (8)	O4^ii^—K2—O7^xii^	95.32 (5)
O3^ix^—Fe1—O3^iii^	180.00 (5)	O6^vi^—K2—O7^xii^	125.92 (6)
O2—P1—O1	110.92 (8)	O1^i^—K2—O7^xii^	56.34 (4)
O2—P1—O4	114.19 (8)	O4—K2—O2	52.08 (5)
O1—P1—O4	108.73 (8)	O6^xii^—K2—O2	125.05 (6)
O2—P1—O3	108.41 (8)	O2^i^—K2—O2	56.55 (6)
O1—P1—O3	112.61 (8)	O5^i^—K2—O2	87.27 (5)
O4—P1—O3	101.72 (8)	O4^ii^—K2—O2	59.33 (5)
O7—P2—O6	113.84 (12)	O6^vi^—K2—O2	120.57 (5)
O7—P2—O5^x^	112.26 (7)	O1^i^—K2—O2	56.94 (4)
O6—P2—O5^x^	104.37 (7)	O7^xii^—K2—O2	113.14 (5)
O7—P2—O5	112.26 (7)	O7^xi^—K3—O5	139.0 (2)
O6—P2—O5	104.37 (7)	O7^xi^—K3—O5^x^	139.0 (2)
O5^x^—P2—O5	109.14 (11)	O5—K3—O5^x^	55.61 (11)
O3—K1—O3^x^	72.22 (7)	O7^xi^—K3—O6^xi^	55.72 (11)
O3—K1—O2	56.19 (4)	O5—K3—O6^xi^	143.7 (2)
O3^x^—K1—O2	125.17 (6)	O5^x^—K3—O6^xi^	143.7 (2)
O3—K1—O2^x^	125.17 (6)	O7^xi^—K3—O6^xiii^	149.1 (3)
O3^x^—K1—O2^x^	56.19 (4)	O5—K3—O6^xiii^	65.77 (12)
O2—K1—O2^x^	147.77 (10)	O5^x^—K3—O6^xiii^	65.77 (12)
O3—K1—O3^ix^	138.56 (8)	O6^xi^—K3—O6^xiii^	93.4 (2)
O3^x^—K1—O3^ix^	93.79 (6)	O7^xi^—K3—O2	80.84 (14)
O2—K1—O3^ix^	136.75 (7)	O5—K3—O2	59.12 (9)
O2^x^—K1—O3^ix^	67.30 (4)	O5^x^—K3—O2	105.29 (19)
O3—K1—O3^ii^	93.79 (6)	O6^xi^—K3—O2	110.40 (11)
O3^x^—K1—O3^ii^	138.56 (8)	O6^xiii^—K3—O2	114.13 (11)
O2—K1—O3^ii^	67.30 (4)	O7^xi^—K3—O2^x^	80.84 (14)
O2^x^—K1—O3^ii^	136.75 (7)	O5—K3—O2^x^	105.29 (19)
O3^ix^—K1—O3^ii^	70.89 (7)	O5^x^—K3—O2^x^	59.12 (9)
O3—K1—O5	115.32 (5)	O6^xi^—K3—O2^x^	110.40 (11)
O3^x^—K1—O5	155.60 (8)	O6^xiii^—K3—O2^x^	114.13 (11)
O2—K1—O5	59.36 (5)	O2—K3—O2^x^	112.8 (2)
O2^x^—K1—O5	106.12 (6)		

Symmetry codes: (i) −*x*+1/2, −*y*+3/2, −*z*+1; (ii) −*x*+1, *y*, −*z*+1; (iii) *x*, *y*, *z*−1; (iv) −*x*+1, *y*, −*z*+2; (v) *x*+1/2, −*y*+3/2, *z*+1; (vi) *x*+1/2, *y*+1/2, *z*+1; (vii) −*x*+1, −*y*+1, −*z*; (viii) *x*, −*y*+1, *z*−1; (ix) −*x*+1, −*y*+1, −*z*+1; (x) *x*, −*y*+1, *z*; (xi) *x*, *y*, *z*+1; (xii) −*x*+1/2, −*y*+3/2, −*z*; (xiii) −*x*, −*y*+1, −*z*; (xiv) *x*, −*y*+2, *z*.
